# Supplementary material for: Prevalence and factors associated with early resumption of sexual intercourse among postpartum women: Systematic review and meta-analysis
Source: PLoS One. 2024 Jan 17;19(1):e0288536. doi: 10.1371/journal.pone.0288536 (PMC10793940; doi:10.1371/journal.pone.0288536)
Supplement: S2 Table — (DOCX) [file pone.0288536.s002.docx]

Supplementary 2. Quality assessment for the included Studies

| Item | Clearly defined inclusion | Describe the study setting and participant | Valid and reliable exposure measurement | Objective and standard criteria for measurement | Identified confounder | Strategies to deal with confounders | | Valid and reliable outcome measurement | | Appropriate static analysis | | | No of ‘yes’s ‘ |
| --- | --- | --- | --- | --- | --- | --- | --- | --- | --- | --- | --- | --- | --- |
| Xiaorong Fan et.al | Yes | Yes | No | Yes | Yes | No | | Yes | | Yes | | | 6/8=75 |
| Caixia Zhuang et.al | Yes | Yes | Yes | Yes | No | No | | Yes | | Yes | | | 6/8=75 |
| Prakash P et.al | Yes | Yes | No | Yes | Yes | Yes | | Yes | | Yes | | | 7/8=87.5 |
| Gyan P et.al | Yes | Yes | Yes | Yes | Yes | No | | Yes | | Yes | | | 7/8=87.5 |
| Fatemeh D et.al | Yes | Yes | No | Yes | Yes | Yes | | Yes | | Yes | | | 7/8=87.5 |
| Olugbenga Bello et.al | Yes | Yes | No | Yes | Yes | No | | Yes | | Yes | | | 6/8=75 |
| Anzaku AS eta.al | Yes | Yes | Yes | Yes | Yes | No | | Yes | | Yes | | | 7/8=87.5 |
| Kola M | Yes | Yes | Yes | Yes | No | No | | Yes | | Yes | | | 6/8=75 |
| Alice C et. al/ | Yes | Yes | No | Yes | Yes | Yes | | Yes | | Yes | | | 7/8=87.5 |
| MADENJE M et.al | Yes | Yes | Yes | Yes | No | No | | Yes | | Yes | | | 6/8=75 |
| Emmanuel O et.al | Yes | Yes | Yes | Yes | Yes | No | | Yes | | Yes | | | 7/8=87.5 |
| Tariku B et.al | Yes | Yes | Yes | Yes | Yes | No | | Yes | | Yes | | | 7/8=87.5 |
| Dejene E/ et.al | Yes | Yes | Yes | Yes | Yes | No | | Yes | | Yes | | | 7/8=87.5 |
| Melaku H et.al | Yes | Yes | No | Yes | Yes | No | | Yes | | Yes | | | 6/8=75 |
| Ebisa Turi et.al | Yes | Yes | Yes | Yes | Yes | Yes | | Yes | | Yes | | | 7/8=75 |
| Frewoini T/2014 | Yes | Yes | No | Yes | Yes | Yes | | Yes | | Yes | | | 7/8=85.5 |
| **For case-control** | | | | | | | | | | | | | |
|  | | | | | | | | | | | | | |
| Criteria | Sònia Anglès et.al | | | Kathrine Fodstad et.al | | | |  |  |  |  |  |  |
| Compilations of cases and control | Yes | | | Yes | | | |  |  |  |  |  |  |
| Matched case and controls | Yes | | | No | | | |  |  |  |  |  |  |
| The same criteria used to identify case and control | Yes | | | Yes | | | |  |  |  |  |  |  |
| Standard, valid, and reliable measurement of exposure | Yes | | | No | | | |  |  |  |  |  |  |
| The same measurement of exposure for case and control | Yes | | | Yes | | | |  |  |  |  |  |  |
| Identifying confounder | No | | | No | | | |  |  |  |  |  |  |
| Strategies to identify cofounders | Yes | | | Yes | | | |  |  |  |  |  |  |
| Standard, valid, and reliable ways to assess outcomes for case and control | Yes | | | Yes | | | |  |  |  |  |  |  |
| Long enough period of exposure | No | | | Yes | | | |  |  |  |  |  |  |
| Appropriate statistical analysis | Yes | | | Yes | | | |  |  |  |  |  |  |
| Percentage of yes (%) | 8/10=80% | | | 7/10=70% | | | |  |  |  |  |  |  |
| **For prospective cohort** | | | | | | | | | | | | | |
| Criteria | | | | | Yee L et.al | | | | Brubaker L et.al | | Rose N et.al | | |
| The two groups are similar and recruited from the same population | | | | | Yes | | | | Yes | | Yes | | |
| Similar measurements of exposure both for exposed and  unexposed groups | | | | | Yes | | | | Yes | | Yes | | |
| Valid and reliable measurement of exposure | | | | | Yes | | | | Yes | | Yes | | |
| Identifying confounders | | | | | Yes | | | | Yes | | Yes | | |
| Strategies to deal with confounders | | | | | No | | | | Yes | | Yes | | |
| Groups are free of the outcomes at the beginning | | | | | Yes | | | | Yes | | No | | |
| Valid and reliable measurement of outcomes | | | | | Yes | | | | Yes | | Yes | | |
| Long enough follow-up time for the occurrence of outcomes | | | | | Yes | | | | No | | Yes | | |
| Complete follow-up time | | | | | Yes | | | | Yes | | Yes | | |
| Strategies to address lost follow-up | | | | | No | | | | No | | | | |
| Percentage of yes (%) | | | | | 8/10=80% | | 8/10=80% | | | | | 9/10/90% | |
