# Supplementary material for: Prevalence and factors associated with early resumption of sexual intercourse among postpartum women: Systematic review and meta-analysis
Source: PLoS One. 2024 Jan 17;19(1):e0288536. doi: 10.1371/journal.pone.0288536 (PMC10793940; doi:10.1371/journal.pone.0288536)
Supplement: Attachment — Submitted filename: Review comments by Mohammed S.docx [file pone.0288536.s003.docx]

Review comments by Mohammed S. Obsa

Factors associated with early resumption of sexual intercourse among postpartum.

women: Systematic review and meta-analysis

Write a step-by-step response to these comments:

- The area of the study is very important; however, the manuscript needs major revision to be accepted for publications in the PLoS one.
- It is essential that a manuscript should undergo gross language editing before it is accepted for publication in PLoS one.
- Include the total sample size of this study in the abstract as well.
- Explain briefly why you used statistical methods in your abstracts for major findings.
- This conclusion does not seem to make sense, so it should be refined.
- Would you recommend resuming early sexual relations?
- The introduction should begin with a brief description of the study's background. The introduction in this case was not focused.
- In the introduction, the author should describe the magnitude of the problem and what factors affect it.
- This gap was not clearly identified by the author, and it would be helpful if they could specify where it lies?
- There should be a thorough mention of the key term used for the search. The manuscript presents search terms inconsistently.
- In both cases, you mention exclusion criteria, but they are contradictory. It is therefore necessary to revise it thoroughly. It is recommended that the exclusion criteria be the default inclusion criteria.
- The author should use a citation software programme like Endnote. It is evident from what is written in the characteristics of the included studies that poor citation styles have been applied.
- The order in which the results were presented was inappropriate. After exploring sources of heterogeneity, publication bias should be investigated.
  - The author should check the assumptions for subgroup analysis before running the data.
  - The discussion should be substantially revised. There is a lack of coherence and implications of the major findings in the paper.
- It is recommended that the order of discussion follow the order of importance of the variables.
- There should be a clear explanation of the strengths and limitations of this review.
