# Supplementary material for: Prevalence and factors associated with early resumption of sexual intercourse among postpartum women: Systematic review and meta-analysis
Source: PLoS One. 2024 Jan 17;19(1):e0288536. doi: 10.1371/journal.pone.0288536 (PMC10793940; doi:10.1371/journal.pone.0288536)
Supplement: Attachment — Submitted filename: Response for reviewer.docx [file pone.0288536.s005.docx]

Point by point response for reviewer

**Reviewer #1:**

I think that Authors should clarify some points and improve the quality of manuscript citing relevant and novel key articles about the topic:
-the role of the perineum protection techniques during the management of the second phase of labour and the effect on the postpartum period (authors may refer to: PMID: 25909491; PMID: 24942141).
I think that this modification make the work more complete. . Considered all these points, I think it could be of interest for the readers and, in my opinion, it deserves the priority to be published after minor revisions.

**Author response: we have updated as following**

The previous study has provided evidence that the use of hands-free perineal control techniques during the second stage of labor may represent a promising delivery approach to maintain perineal integrity. In addition, these techniques can have a positive effect on the resumption of sexual activity in the postpartum period, as they include a number of factors that can facilitate this process [14]. Numerous cohort studies have shown that women who undergo spontaneous vaginal delivery with an intact perineum have a higher likelihood of having vaginal intercourse again within six to eight weeks after birth compared to women who undergo an episiotomy assisted vaginal birth etc. undergo a Cesarean Section[15,16,17].

**References**

14.Laganà AS, Burgio MA, Retto G, Pizzo A, Granese R, Sturlese E, Ciancimino L, Chiofalo B, Retto A, Triolo O. Management of the second phase of labour: perineum protection techniques. Minerva Ginecol. 2015 Jun 1;67(3):289-96.

15. Laganà AS, Burgio MA, Ciancimino L, Sicilia A, Pizzo A, Magno C, Butticè S, Triolo O. Evaluation of recovery and quality of sexual activity in women during postpartum in relation to the different mode of delivery: a retrospective analysis. Minerva Ginecol. 2015 Aug 1;67(4):315-20.

16. McDonald, E.; Brown, S. Does method of birth make a difference to when women resume sex after childbirth? BJOG Int. J. Obstet. Gynaecol. 2013, 120, 823–830. [Google Scholar] [CrossRef]

**Additional Editor Comments:**

Thank you for the resubmission of your study, which the reviewers still read with great interest. We thank the reviewers for their time and contributions to strengthen this body of work. Indeed, there is 1 issue that must be addressed pertaining to the current references cited. Please include updates references that better highlight the gap in knowledge that necessitated conducting this study and the importance of perineum protection techniques during the timing of management during labor.

**Author response: We have updated**

The previous study has provided evidence that the use of hands-free perineal control techniques during the second stage of labor may represent a promising delivery approach to maintain perineal integrity. In addition, these techniques can have a positive effect on the resumption of sexual activity in the postpartum period, as they include a number of factors that can facilitate this process [14]. Numerous cohort studies have shown that women who undergo spontaneous vaginal delivery with an intact perineum have a higher likelihood of having vaginal intercourse again within six to eight weeks after birth compared to women who undergo an episiotomy assisted vaginal birth etc. undergo a Cesarean Section[15,16,17].

Therefore, it is important to examine the various aspects of early resumption of sexual relationships in the postpartum period to understand the direct and indirect effects of this problem and highlight the importance of studying these issues in global level [19].
